# A pilot randomized controlled trial of automated and counselor-delivered text messages for e-cigarette cessation

**DOI:** 10.18332/tpc/157598

**Published:** 2023-02-14

**Authors:** Nandita Krishnan, Carla J. Berg, Daisy Le, Jasjit Ahluwalia, Amanda L. Graham, Lorien C. Abroms

**Affiliations:** 1 Department of Prevention and Community Health, Milken Institute School of Public Health, George Washington University, Washington, United States; 2 George Washington Cancer Center, George Washington University, Washington, United States; 3 Department of Policy, Populations and Systems, George Washington University School of Nursing, Washington, United States; 4 Department of Behavioral and Social Sciences and Center for Alcohol and Addiction Studies, Brown University School of Public Health, Rhode Island, United States; 5 Innovations Center, Truth Initiative, Washington, United States; 6 Department of Medicine, Mayo Clinic College of Medicine and Science, Rochester, United States

**Keywords:** e-cigarette, cessation, text messaging

## Abstract

**INTRODUCTION:**

Automated text messaging programs show promise for e-cigarette cessation. Adding live text counseling could make them more engaging. We developed Quit the Vape (QTV), an automated e-cigarette cessation text messaging program, designed to be delivered as stand-alone or with counselor-delivered messages (QTV-C), and evaluated the acceptability and preliminary efficacy of QTV and QTV-C.

**METHODS:**

Between May and August 2021, we recruited 58 e-cigarette users, aged 20–43 years, 53.5% male, 63.8% non-Hispanic White, from an ongoing cohort study in the United States. Inclusion criteria were: using nicotine-containing e-cigarettes on ≥4 days per month; smartphone ownership; and not receiving tobacco cessation treatment. Motivation to quit did not impact eligibility. Participants were randomized to QTV (n=20), QTV-C (n=19), or control (link to e-cigarette cessation website, n=19). At end-of-treatment, we assessed program engagement and satisfaction, and self-reported quitting behaviors (e.g. point prevalence abstinence, PPA).

**RESULTS:**

At baseline, average past-month e-cigarette use was 26.8 days (SD=6.2). At follow-up at 4 weeks, among QTV and QTV-C participants, ≥85% replied to ≥1 message, ≥35% set a quit date, and ≤15% opted out. More QTV and QTV-C participants (55.6%) versus control (17.7%) reported program satisfaction (p=0.034). QTV-C participants (vs QTV and control) trended more favorably on 7-day e-cigarette PPA [27.8% (95% CI: 11.5–53.3) vs 11.1% (95% CI: 2.6–37.0) and 5.9% (95% CI: 0.7–34.5)] and quit attempts [66.7% (95% CI: 41.6–84.9) vs 50.0% (95% CI: 27.4–72.6) and 52.9% (95% CI: 29.2–75.5)].

**CONCLUSIONS:**

Adding live text counseling to an automated text messaging program is acceptable and shows promise for e-cigarette cessation. A larger trial is warranted to assess its efficacy.

## INTRODUCTION

E-cigarettes are currently the second most commonly used tobacco product among adults (3.7%; 9.1 million people) in the US^1^. A growing body of evidence indicates that e-cigarettes deliver fewer harmful chemicals than traditional cigarettes^2,3^ and may support smoking cessation^4^; thus, e-cigarettes may offer a harm reduction benefit for people who smoke cigarettes, provided they completely switch to using e-cigarettes. However, e-cigarettes pose independent health risks including nicotine addiction^5,6^ and lung and heart disease^7,8^, and their long-term effects are unknown^3^. Considering these risks, e-cigarettes are not recommended for adults who do not currently use tobacco products^9^; nevertheless, use in this group has increased^10^.

The majority (>60%) of adults who use e-cigarettes plan to quit^11,12^. However, e-cigarette cessation intervention research has not kept pace with the demand for cessation assistance. The current evidence-base for e-cigarette cessation interventions is largely limited to youth and young adults, and consists of an observational study of treatment-seeking teens and young adults (n=27000)^13^; several clinical case reports^14-17^; a single-arm pilot study (n=8)^18^; 2 pilot randomized controlled trials (RCT) (n=24; n=27)^19,20^; and one fully powered randomized controlled trial (RCT) of an automated text message vaping cessation intervention (n=2588)^21^. The fully powered RCT reported higher e-cigarette abstinence rates among young adults (18–24 years) randomized to the intervention than Control (24.1% vs 18.6%) at 7 months post randomization^21^.

While automated text messages show promise for e-cigarette cessation^21^ and have an established evidence-base for cigarette smoking cessation^22^, some users find these programs impersonal and prefer being able to interact with a real person^23,24^. Automated programs can be enhanced by incorporating human-delivered counseling, and emerging evidence from text messaging and social media-based interventions suggests that this hybrid approach may be more effective and engaging than exclusively automated programs^25-27^. Considering the nuances of e-cigarette use, including co-use with other substances, and varying motives for use and levels of readiness to quit^28^, the addition of human-delivered counseling to automated text messages could provide more intensive, personalized support to help people who use e-cigarettes to successfully quit.

The objective of this pilot study was to examine the acceptability and preliminary efficacy of Quit the Vape (QTV), an automated text messaging program for e-cigarette cessation, delivered with and without live text counseling. Specifically, participants were randomized to one of 3 groups: QTV, QTV + text messages delivered by a counselor (QTV-C), or a control group, that received a single text message referral to an e-cigarette cessation website. At the follow-up at 4 weeks, we examined program engagement and satisfaction, and self-reported e-cigarette quit rates, quit attempts, and frequency of e-cigarette use.

## METHODS

### Sample

Participants were recruited between May and August 2021, from an ongoing cohort study examining vape product use and retail among adults, who were aged 18–34 years when they were recruited via social media in Fall 2018^29^. The sampling frame for this pilot was constructed as follows: participants in the cohort reporting past 30-day e-cigarette use at wave 5 (Fall 2020, n=622) were stratified by past 30-day e-cigarette use levels (i.e. used 30 days; used 21–29 days; used 11–20 days; used ≤10 days). Participants in the first 3 categories (i.e. used 30 days; 21–29 days; or 11–20 days) were randomly selected to participate in the current pilot study. Inclusion criteria were: 1) used nicotine-containing e-cigarettes on ≥4 days within the past 30 days; 2) had a smartphone; and 3) not currently receiving tobacco cessation treatment. Participants who were pregnant were excluded. Cigarette smoking status and motivation to quit using e-cigarettes did not impact eligibility.

### Study procedures

An email invitation with a link to the screener and consent form was sent to selected participants. The invitation indicated that participants were being contacted because they reported using e-cigarettes in the wave 5 survey of the cohort study and that they might be eligible for the present study. Participants were provided a brief description of the study. Those who were eligible and provided consent were automatically routed to an online baseline survey administered via RedCap^30,31^. Participants who completed the baseline survey received a text message invitation to receive messages about quitting e-cigarette use. Screener and text message non-responders received up to 3 additional reminders that were sent for 3 consecutive days following the initial invitation.

Participants who replied yes to the invitation text message were enrolled and randomized to one of 3 groups: QTV, QTV-C, or control, using the REDCap^30,31^ randomization module. Given the small sample size, we used a block randomization scheme with block sizes of 3 and 6 to ensure balanced allocation across study groups. Once participants were randomized, they received messages from their assigned program. Four weeks later, participants received an email with a link to the follow-up survey. Non-responders received up to 3 additional reminders for 3 consecutive days following the initial invitation. Participants received a $10 e-gift card for completing the baseline survey and a $20 e-gift card for completing the follow-up survey.

### Interventions

Participants in the control group received a single text message with a link to the National Cancer Institute’s Smokefree.gov e-cigarette cessation resource (https://teen.smokefree.gov/quit-vaping). QTV and QTV-C participants received automated text messages for 4 weeks.

*Automated text messages*

The messaging framework was adapted from an evidence-based smoking cessation text messaging program^32^. Additionally, messages were informed by qualitative research with adults who use e-cigarettes, a brief review of the literature and medical websites, and searches of online e-cigarette cessation forums (e.g. Reddit e-cigarette cessation groups). Messages were based on social cognitive theory (SCT) principles and aimed at increasing motivation to quit by: highlighting the harms of e-cigarettes and benefits of quitting; building self-efficacy and behavioral capability to quit using e-cigarettes; and providing social support; and helping users deal with cravings, triggers and withdrawal (sample messages are given in Supplementary file Table 1). Messages did not cover dual use, but some messages conveyed harms associated with nicotine use. Some text messages included animated images, such as GIFs and stickers (Supplementary file Figure 1).

Participants were encouraged to set a quit date within 2 weeks of joining the program and were regularly prompted to set a quit date throughout the program. Different messaging protocols were developed for participants who did and did not set a quit date. While messages in both protocols covered all key SCT constructs, the quit date protocol focused more on messages aimed at building self-efficacy and behavioral capability to quit, and tips to deal with cravings, triggers, and withdrawal; the no quit date protocol messages placed more emphasis on building motivation to quit by highlighting harms of e-cigarette use and benefits of quitting. Participants could also text the following on-demand keywords any time: WHY – to read reasons to quit using e-cigarettes; CRAVE – for tips to manage cravings; DATE – to set a quit date; and VAPED – for help to get back on track if they relapsed. QTV and QTV-C participants received about 2 messages/day for the first week, 1 message/day for the second week, and 3 messages/week for the last 2 weeks. Participants who set a quit date received up to 3 messages/day on and around their quit date, with message frequency subsequently tapering off.

*Counselor outreach text messages*

In addition to the automated messages, QTV-C participants also received proactive outreach text messages from a counselor about 2 times/week. Messages from the counselor were prefaced with the counselor’s name, so that participants could distinguish between automated and counselor outreach messages. One of these outreach messages was a generic check-in sent to all participants in the counseling group, and the second message was tailored based on the participant’s baseline survey data (e.g. lives with an e-cigarette user) or engagement with the program (Supplementary file Table 1). Participants who set a quit date received additional counselor outreach messages on and around their quit date. Participants could also text the counselor at any time and receive a response within 24 hours. Text counseling was delivered by a doctoral level student who had received tobacco dependence treatment specialist training.

### Measures

*Baseline characteristics*

Sociodemographics

We included age, gender (male, female, non-binary/third-gender), race/ethnicity (non-Hispanic White, other); and full-time employment (yes, no).

E-cigarette use characteristics

We included days used nicotine-containing e-cigarettes in the past 30 days; e-cigarette dependence, measured using the e-cigarette Fagerström test for nicotine dependence (e-FTND)^33^; past year quit attempt (yes, no); device type (disposable, pre-filled cartridge, refillable tank, mod system); age started using nicotine containing e-cigarettes regularly, and use of flavors (tobacco, menthol/mint, fruit, coffee/tea, alcoholic drinks, caramel/vanilla/chocolate cream, candy, other food).

Smoking and substance use characteristics

We assessed past 30-day use of cigarettes, marijuana, and alcohol. Responses were dichotomized based on any past 30-day use (yes, no).

Psychosocial and environmental characteristics

We assessed readiness to quit using e-cigarettes within the next 6 months (ready, not ready); confidence to quit using e-cigarettes (scale: 0–10); perceived addictiveness and perceived harm of e-cigarettes (scales: 1–7); and depression, using the 4-item Patient Health Questionnaire (PHQ)^34^. Environmental characteristics included whether the participant lives with an e-cigarette user (yes, no), or smoker (yes, no).

*Outcomes*

Program engagement

Engagement data were obtained from the text messaging program metadata for QTV and QTV-C participants. Engagement measures included the proportion of participants who: replied to at least 1 message, set a quit date, and opted out of the program. We also assessed number of interactions with the program, and number of interactions with the counselor (for QTV-C participants). Interactions were defined as any incoming text message from the user.

Program satisfaction

Measures of satisfaction included the proportion of participants who read all/almost all messages, and the following items (assessed using a 5-point Likert scale): whether the messages gave good ideas for quitting e-cigarette use, were helpful, were too frequent, and were a trigger; whether the participant would recommend the program to others; and overall satisfaction with the program. We also assessed satisfaction using the System Usability Scale (SUS) score, a continuous measure^35^, and a score >68 is considered above average. SUS items and scoring are given in Supplementary file Table 2 (A CONSORT checklist for reporting a randomized trial is given in the Supplementary file Table 3).

For QTV-C participants, counselor satisfaction was assessed using the following items (measured on a 5-point Likert scale): whether the counselor responded promptly, was supportive, gave helpful suggestions, and enhanced the program; whether it was clear that there was a counselor and which messages were automated versus from the counselor; and overall satisfaction with the counselor. A rating of 4 or 5 for Likert scale items was coded as satisfied, and the proportion of participants expressing satisfaction for each measure was calculated.

E-cigarette use

Self-reported 7-day point prevalence abstinence (PPA) was assessed at follow-up by asking: ‘On how many days of the past 7 days have you vaped nicotine, even a puff?’. Participants reporting 0 days were considered abstinent from e-cigarette use. Also assessed were quit attempts (defined as stopping the use of nicotine containing e-cigarettes for 24 hours or longer since joining the study because the participant was trying to quit for good) and changes in days used e-cigarettes in the past 30 days between follow-up and baseline.

Cigarette use

Self-reported 7-day PPA was assessed at follow-up by asking: ‘On how many days of the past 7 days have you smoke cigarettes, even a puff?. Participants reporting 0 days were considered abstinent from smoking. For baseline past 30-day cigarette smokers, smoking quit attempts and changes in days used cigarettes in the past 30 days between follow-up and baseline, were also assessed.

### Statistical analysis

Baseline descriptive characteristics were compared across study groups using chi-squared tests for categorical variables and one-way ANOVA for continuous variables to examine differences in groups at randomization. Next, similar analyses were conducted to examine outcomes across intervention groups. Acknowledging that this pilot study was not statistically powered to detect differences in behavioral outcomes between groups, means or proportions along with 95% confidence intervals (CIs) were estimated for e-cigarette and smoking related outcomes^36^. For baseline past 30-day cigarette users, smoking outcomes at follow-up were also explored. Program engagement data was available for all participants. Self-reported outcomes, including e-cigarette and cigarette use were calculated using complete-case analysis. We used this approach rather than intention-to-treat, as transitions between e-cigarette and cigarette use are common^37^; using the standard convention of assuming missing is equivalent to using, would require making several assumptions about these transitions (e.g. baseline dual users continue to use both products at follow-up). All analyses were conducted using Stata 14.

## RESULTS

**Figure 1:**
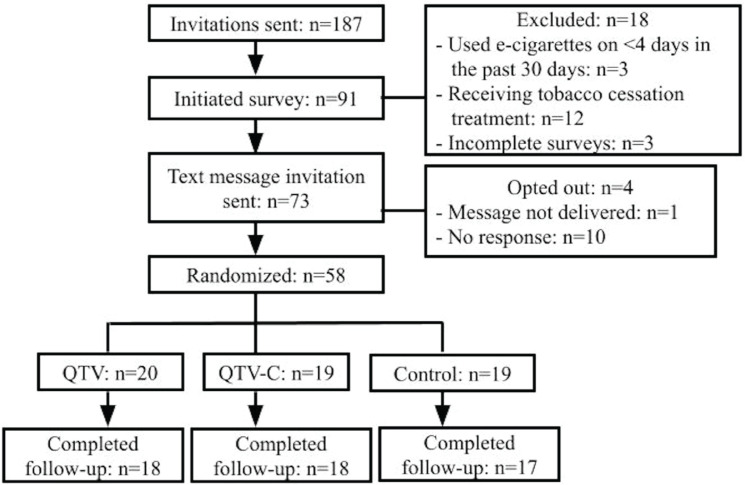
Participant flowchart

Of 187 participants invited to join the study, 73 were eligible, consented to participate, completed the baseline survey, and received the text message invitation to join the program (Figure 1). A total of 58 participants (79.5%) responded to the invitation and were randomized (QTV, n=20; QTV-C, n=19; control, n=19), with high follow-up completion rates at end-of-treatment [QTV, n=18 (90.0%); QTV-C, n=18 (94.7%); control, n=17 (89.5%)].

Participant characteristics are presented in Table 1. Participants had a mean age of 27.3 years (SD=5.5). The majority of participants were male (53.5%) and non-Hispanic White (63.8%). On average, participants used e-cigarettes on 26.8 days per month (SD=6.2). At baseline, the majority of participants (70.2%) reported past 30-day alcohol use, and 41.4% and 42.1% reported past 30-day cigarette and cannabis use, respectively. Almost half the sample (46.6%) was planning to quit using e-cigarettes within 6 months, and 36.2% made a past-year quit attempt. There were no differences in participant characteristics across study groups at baseline, except for PHQ-scores, which were significantly higher for QTV (vs QTV-C).

**Table 1:** Baseline characteristics of participants by study group (N=58)

*Characteristics*	*Total (n=58) n (%)*	*QTV (n=20) n (%)*	*QTV-C (n=19) n (%)*	*Control (n=19) n (%)*
**Sociodemographics**				
**Age** (years), mean ± SD	27.3 ± 5.5	26.6 ± 6.1	28.1 ± 5.3	27.4 ± 5.0
**Gender**				
Male	31 (53.5)	8 (40.0)	11 (57.9)	12 (63.2)
Female	24 (41.4)	10 (50.0)	8 (42.1)	6 (31.6)
Non-binary/third gender	3 (5.2)	2 (10.0)	0 (0.0)	1 (5.3)
**Non-Hispanic White**	37 (63.8)	15 (75.0)	14 (73.7)	8 (42.1)
**Employed full-time**	37 (63.8)	12 (60.0)	11 (57.9)	14 (73.7)
**E-cigarette use characteristics**				
Days used nicotine containing e-cigarettes in last 30 days, mean ± SD	26.8 ± 6.2	26.5 ± 6.0	26.5 ± 7.5	27.6 ± 5.4
e-FTND score (0–10), mean ± SD	4.6 ± 2.3	4.9 ± 2.3	4.2 ± 2.4	4.7 ± 2.2
Past year e-cigarette quit attempt	21 (36.2)	6 (30.0)	8 (42.1)	7 (36.8)
**Device type**				
Disposable	8 (13.8)	2 (10.0)	3 (15.8)	3 (15.8)
Pre-filled cartridges	19 (32.8)	5 (25.0)	8 (42.1)	6 (31.6)
Refillable tank	23 (39.7)	9 (45.0)	7 (36.8)	7 (36.8)
Mod system	8 (13.8)	4 (20.0)	1 (5.3)	3 (15.8)
**Age started using nicotine-containing e-cigarettes regularly,** mean ± SD	22.9 ± 5.6	22.1 ± 5.4	24.0 ± 5.9	22.8 ± 5.8
**Top 3 flavors used^a^**				
Fruit	40 (69.0)	14 (70.0)	13 (68.4)	13 (68.4)
Menthol/mint	34 (58.6)	13 (65.0)	10 (52.6)	11 (57.9)
Candy	17 (29.3)	5 (25.0)	6 (31.6)	6 (31.6)
**Smoking and substance use characteristics**				
Past 30-day cigarette use	24 (41.4)	10 (50.0)	8 (42.1)	6 (31.6)
Past 30-day cannabis use^b^	24 (42.1)	6 (30.0)	10 (52.6)	8 (44.4)
Past 30-day alcohol use^b^	40 (70.2)	12 (60.0)	15 (79.0)	13 (72.2)
**Psychosocial and environmental characteristics**				
Planning to quit using e-cigarettes within 6 months	27 (46.6)	10 (50.0)	10 (52.6)	7 (36.8)
Confidence to quit using e-cigarettes (0–10), mean ± SD	5.6 ± 2.6	5.1 ± 2.7	6.1 ± 2.4	5.8 ± 2.8
Perceived addictiveness of e-cigarettes (1–7), mean ± SD	5.7 ± 1.5	5.3 ± 1.7	5.9 ± 1.0	6.1 ± 1.6
Perceived harm of e-cigarettes (1–7), mean ± SD	4.3 ± 1.5	4.2 ± 1.4	4.5 ± 1.3	4.3 ± 1.8
Perceived social acceptability of e-cigarettes (1–7), mean ± SD	5.4 ± 1.5	5.8 ± 1.2	5.0 ± 1.6	5.5 ± 1.8
Lives with an e-cigarette user^c^	22 (40.0)	8 (40.0)	7 (41.2)	7 (38.9)
Lives with a smoker^c^	19 (34.6)	6 (30.0)	6 (35.3)	7 (38.9)
PHQ score (0–12), mean ± SD^d^	4.5 ± 3.7	6.2 ± 4.5	3.4 ± 2.8	3.8 ± 2.9

a Participants could select more than one flavor.

b Data missing for n=1.

c Data missing for n=3.

d Post-hoc comparisons using Bonferroni correction indicated that PHQ score significantly differed for QTV vs QTV-C participants. e-FTND: Fagerströmtest for nicotine dependence adapted for e-cigarette use. PHQ: patient health questionnaire. QTV: quit the vape. QTV-C: quit the vape with counselor-delivered messages. SD: standard deviation.

Program engagement and satisfaction are shown in Table 2. Opt-out rates were low across all programs (8.6%). Among QTV participants, 85% (17 in 20; 17/20) replied to at least 1 text message, 35% (7/20) set a quit date, and 15% (3/20) opted out. Among QTV-C participants, 89.5% (17/19) replied to 1 or more messages, 36.8% (7/19) set a quit date, and 5.3% (1/19) opted out. QTV participants interacted an average of 6.3 times [SD=8.6; median (IQR)=3 (1–8)], while QTV-C participants interacted 8.6 times [SD=12.0; median (IQR)=3 (1– 13)] with the program. Among QTV-C participants, 42.1% (8/19) engaged with the counselor. These 8 participants engaged an average of 6.8 times [SD=6.7; median (IQR)=5 (1–10.5)] with the counselor.

**Table 2:** Program satisfaction and engagement at follow-up at 4 weeks, by study group (N=58)

*Program engagement^a^*	*Total (n=58) n (%)*	*QTV (n=20) n (%)*	*QTV-C (n=19) n (%)*	*Control (n=19) n (%)*	*p*
Replied to at least 1 message	-	17 (85.0)	17 (89.5)	na	0.676
Set QD	-	7 (35.0)	7 (36.8)	na	0.905
Opted out	5 (8.6)	3 (15.0)	1 (5.3)	1 (5.3)	0.455
Interactions with program, mean ±SD	-	6.3 ± 8.6	8.6 ± 12.0	na	0.489
Interactions with counselor, mean ± SD	-	na	2.8 ± 5.4	na	-
***Program satisfaction^a^***	***n/N (%)***	***n/N (%)***	***n/N (%)***	***n/N (%)***	***p***
Read all/almost all messages	31/53 (58.5)	10/18 (55.6)	10/18 (55.6)	11/17 (64.7)	0.819
Messages gave good ideas^b^	26/48 (54.2)	12/18 (66.7)	10/18 (55.6)	4/12 (33.3)	0.197
Messages were helpful^b^	24/49 (49.0)	9/18 (50.0)	10/18 (55.6)	5/13 (38.5)	0.639
Messages too frequent^b^	20/49 (40.8)	10/18 (55.6)	8/17 (47.1)	2/14 (14.3)	**0.050**
Messages were a trigger^b^	14/48 (29.2)	8/17 (47.1)	3/18 (16.7)	3/13 (23.1)	0.121
Would recommend the program^b^	26/50 (52.0)	10/18 (55.6)	9/18 (50.0)	7/14 (50.0)	0.931
Overall satisfaction^b^	23/53 (43.4)	10/18 (55.6)	10/18 (55.6)	3/17 (17.7)	**0.034**
System Usability Scale score, mean ± SD	70.9 ± 13.3	72.6 ± 13.9	73.9 ± 13.3	66.0 ± 17.0	0.252
**Counselor satisfaction^a^**					
Responded promptly^b^	-	na	7/10 (70.0)	na	-
Supportive^b^	-	na	9/12 (75.0)	na	-
Gave helpful suggestions^b^	-	na	8/11 (72.7)	na	-
Enhanced the program^b^	-	na	7/11 (63.6)	na	-
Clear there was a real counselor^b^	-	na	10/15 (66.7)	na	-
Clear which messages were automated vs from counselor^b^	-	na	11/15 (73.3)	na	-
Overall satisfaction with counselor^b^	-	na	8/15 (53.3)	na	-

a Program engagement metrics were obtained from the text messaging program metadata, and program and counselor satisfaction were based on self-report at follow-up.

b Refers to those who selected a rating of 4 or 5 on a 5-point Likert scale. na: not applicable. QD: quit date. QTV: quit the vape. QTV-C: quit the vape with counselor-delivered messages. SD: standard deviation.

At follow-up at 4 weeks, a higher proportion of QTV and QTV-C participants (55.6%; 10/18) expressed overall satisfaction with the program relative to control group participants (17.7%; 3/17; p=0.034). Among QTV and QTV-C participants, 66.7% (12/18) and 55.6% (10/18) felt that the messages provided good ideas (vs control: 33.3%; 4/12), and 50.0% (9/18) and 55.6% (10/18) felt that the messages were helpful (vs control: 38.5%; 5/13). More participants in the intervention groups (vs control) felt that the messages were too frequent (p=0.05). Fewer QTV-C participants felt that the messages were a trigger [16.7% (3/18) vs QTV: 47.1% (8/17) and control: 23.1% (3/13)]. Mean SUS scores for both intervention groups were above average (>68)^35^, indicating that both programs were user-friendly. Among QTV-C participants, ≥70% expressed satisfaction with the counselor across the following dimensions: promptness of responses (70%; 7/10), supportiveness (75%; 9/12), and helpfulness of suggestions (72.7%; 8/11).

E-cigarette and smoking outcomes are presented in Table 3. Seven-day e-cigarette PPA was reported by 11.1% (2/18) (95% CI: 2.6–37.0) of QTV, 27.8% (5/18) (95% CI: 11.5–53.3) of QTV-C, and 5.9% (1/17) (95% CI: 0.7–34.5) of control participants. E-cigarette quit attempts were reported by 50.0% (9/18) (95% CI: 27.4–72.6) of QTV, 66.7% (12/18) (95% CI: 41.6–84.9) of QTV-C, and 52.9% (9/17) (95% CI: 29.2–75.5) of control participants. Changes in e-cigarette use frequency also trended toward those in intervention groups reporting greater reductions compared to control. Results also showed a trend toward higher proportions of baseline dual users in both intervention groups achieving 7-day cigarette PPA and greater reductions in days smoked versus control. Among the 22 baseline dual users, 1 participant (in QTV) achieved 7-day PPA for cigarettes and e-cigarettes; 7 participants (31.8%) achieved 7-day PPA for cigarettes only [QTV: 44% (4/9); QTV-C: 37.5% (3/8)].

**Table 3:** E-cigarette and smoking outcomes at follow-up at 4 weeks, by study group

	*Total (n=53) % (95% CI)*	*QTV (n=18) % (95% CI)*	*QTV-C (n=18) % (95% CI)*	*Control (n=17) % (95% CI)*	*p*
**E-cigarette outcomes**					
7-day e-cigarette PPA	15.1 (7.6–27.9)	11.1 (2.6–37.0)	27.8 (11.5–53.3)	5.9 (0.7–34.5)	0.165
Made an e-cigarette quit attempt	56.6 (42.7–69.6)	50.0 (27.4–72.6)	66.7 (41.6–84.9)	52.9 (29.2–75.5)	0.561
Change in days used e-cigarettes in last 30 days,mean (95% CI)^a^	-6.5 (-8.9– -4.0)	-7.6 (-12.6– -2.6)	-6.7 (-11.2– -2.3)	-5.0 (-8.8– -1.2)	0.684
**Smoking outcomes among baseline dual users**					
	***(n=22)***	***(n=9)***	***(n=8)***	***(n=5)***	***p***
7-day cigarette PPA	36.4 (18.2–59.5)	55.6 (19.5–86.6)	37.5 (8.7–79.2)	0	0.117
Made a smoking quit attempt^b^	45.0 (23.8–68.2)	37.5 (8.7–79.2)	37.5 (8.7–79.2)	75.0 (4.1–99.5)	0.403
Change in days smoked in last 30 days, mean (95% CI)^a^	-3.7 (-6.3– -1.0)	-5.2 (-10.9–0.5)	-3.9 (-8.1–0.4)	-0.6 (-4.8–3.6)	0.396

a Calculated as follow-up – baseline.

b Data missing for n=2.

PPA: point prevalence abstinence. QTV: quit the vape. QTV-C: quit the vape with counselor-delivered messages.SD: standard deviation.

## DISCUSSION

In this pilot RCT, a greater proportion of participants who received automated text messages for e-cigarette cessation (with or without counselor-delivered messages) were satisfied with the program relative to control group participants. Both versions of the text messaging program elicited high engagement. E-cigarette quit rates and quit attempts trended more favorably for QTV-C.

Similar to findings of a previous study that compared automated text messages with and without peer mentoring for smoking cessation^25^, we found that overall program engagement and satisfaction did not differ between the two intervention groups. The high engagement for both intervention groups is especially encouraging, given that more than half the sample (53.4%) were not planning to quit within 6 months and this was not a treatment seeking sample. It is also worth noting that fewer QTV-C participants (16.7%) found the messages to be a trigger than QTV participants (47.1%). A prior analysis of user experiences with an automated smoking cessation text messaging program found that a common source of dissatisfaction was that the messages were a trigger for smoking^23^. This finding suggests that the addition of live counseling could potentially mitigate the triggering effect of automated messages by enabling users to get immediate help and support from the counselor to deal with the trigger. Qualitative research with users could further explore this hypothesis and other perceptions of live text counseling.

E-cigarette quit rates and quit attempts trended in favor of QTV-C. Unlike previous studies^21,25^, we included participants regardless of readiness to quit. Future studies could compare these two text messaging approaches among e-cigarette users who are actively thinking about quitting. While the program did not explicitly address cigarette smoking, 7-day cigarette PPA rates and reductions in days smoked among dual users also trended favorably for QTV and QTV-C (vs control). Given that transitions between e-cigarette and cigarette use are common^37^, it is important for e-cigarette cessation intervention studies to monitor cigarette smoking to ensure that e-cigarette users who quit using e-cigarettes do not maintain or initiate cigarette smoking.

### Limitations

This study had some limitations. This pilot was not statistically powered to detect differences between groups in e-cigarette related outcomes. E-cigarette use was not biochemically verified for determining inclusion or assessing abstinence, which could have resulted in non-users joining the study, and misclassification of abstinence. The larger cohort study from which participants were recruited for this trial, recruited participants from 6 metropolitan areas in different regions of the US using social media^29^. Thus, while findings cannot be extrapolated to some populations, such as people who use e-cigarettes living in rural areas, they can be generalized to adults who use e-cigarettes in different metropolitan regions of the US. Additionally, as motivation to quit using e-cigarettes was not an inclusion criterion, findings can be generalized to e-cigarette users with varying levels of motivation to quit. As one of the first studies to use live-text counseling for e-cigarette cessation, this work can guide future models for developing and integrating live text counseling protocols into automated tobacco cessation and behavior change programs.

## CONCLUSIONS

Adding live text counseling to an automated text messaging program is acceptable to adults who use e-cigarettes and shows promise in facilitating e-cigarette cessation and quit attempts. Qualitative research examining participant experiences and perceptions can inform strategies to improve program satisfaction and engagement. A fully powered trial is needed to assess the efficacy of this approach for e-cigarette cessation.

## Data Availability

The data supporting this research are available from the authors on reasonable request.
