# Supplementary material for: A pilot randomized controlled trial of automated and counselor-delivered text messages for e-cigarette cessation
Source: Tob Prev Cessat. 2023 Feb 14;9:04. doi: 10.18332/tpc/157598 (PMC9926687; doi:10.18332/tpc/157598)
Supplement: Supplementary file 1 [file TPC-9-04-s1.pdf]

Supplementary Table 1. Sample automated and counselor outreach text messages

Automated text messages	
Welcome	Welcome to Quit the Vape (QTV)! We'll send automated tips for 1 month to help you quit vaping nicotine. QTV is an automated text messaging program to help you quit vaping. QTV will help you figure out why you're quitting, set a quit date, deal with cravings & triggers, & stay quit. We'll get you ready.<br>Setting a quit date is an important part of quitting. Mondays are good days to try quitting. Reply DATE to set your quit date.
Harms	Think vaping is harmless? Think again. E-juice contains many toxic chemicals, including chemicals found in weed killers and car exhaust
Behavioral capability	Try a Mini-Quit challenge to help you prepare to deal with withdrawal. Try and make it through the next 8 hours without vaping. We'll check back in then.
Social support	Share your resolution to quit vaping on your social media accounts. It can help you stay on track & feel supported!
Managing triggers	You can quit even if your friends or roommates vape. Ask them not to vape around you or give you their vape, even if you beg for it.
Coping with withdrawal	Quitting is hard because your body is adjusting to life without nicotine. Be good to your body by drinking lots of water & getting at least 8 hrs of sleep.
WHY keyword	I quit vaping because I was getting out of breath on the soccer field & I didn't want to let my team down.
CRAVE keyword	Put your e-cig where you can't see it. Remember: out of sight, out of mind!
VAPED keyword	Slips happen. Don't beat yourself up. Throw out your vape, pods or cartridges, & keep going. You can do it! Remember, you can text CRAVE for help when a craving hits.
Counselor outreach text messages	
Welcome	Welcome to Quit the Vape (QTV)! I'm Maya, a trained counselor. I've created a customized plan for you based on your survey responses. You'll get personalized live help from me + automated QTV tips for 1 month to help you quit vaping nicotine. QTV will help you figure out why you're quitting, set a quit date, deal with cravings & triggers, & stay quit.<br>I'll be reaching out to check on you. You can also text me anytime by replying to any msg from the program & I'll try to reply within 24 hrs. Try it now! Text me for tips on picking a quit date or other questions you have about quitting. Then, text DATE to set your quit date.
Generic outreach	Hi [participant name], I'm checking in to see how you're doing. Text me back and let me know! I'm here for the next 2 hours.
Tailored outreach based on baseline survey response	You can quit vaping even if you live with someone who vapes. Talk to them about making your home vape-free or making common areas vape-free. Having these conversations may seem uncomfortable, but it doesn't have to be. Text me for ideas on how to start the conversation.
Tailored outreach based on use of program features	Hi [participant name], I see that you've read some reasons to quit. Would you like to share your reason with me?
Tailored outreach for participants not engaging with the program	Hi [participant name], I noticed you haven't tried out any QTV keywords. You can text WHY to read reasons people have for quitting. Or I can tell you my favorites. Some of them may surprise you! Give it a try & text me back with your reason!
Tailored outreach around quit date	Hi [participant name], Happy Quit Day! Just checking in to see how it's going & if there's anything you need help with?

Supplementary Figure 1. Sample animated messages

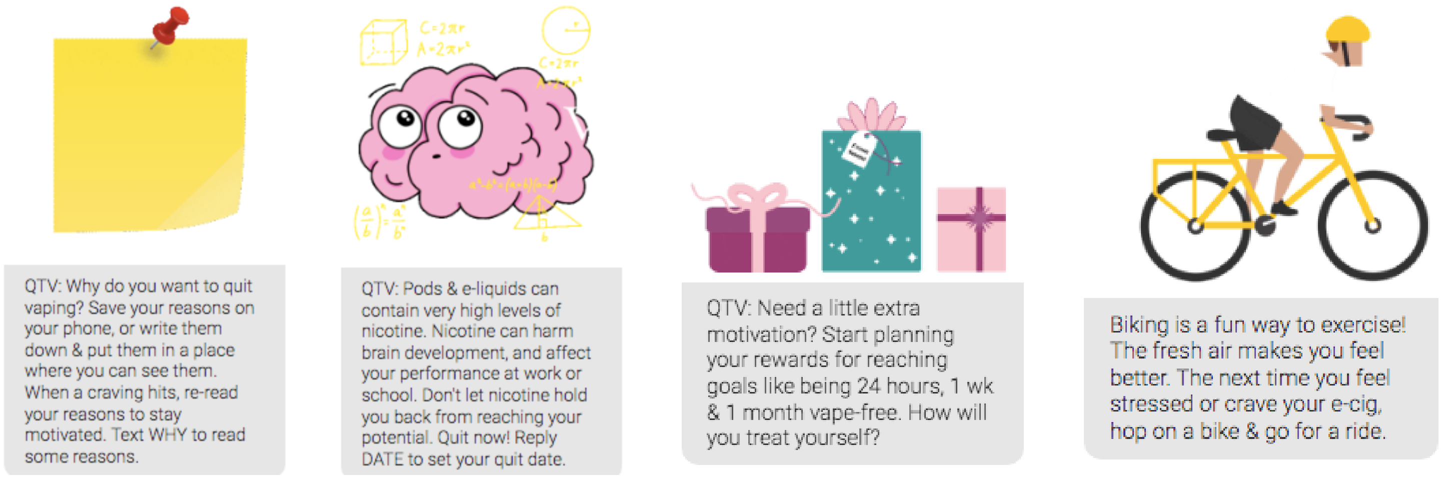

Supplementary Table 2. System Usability Scale (SUS) scoring

Items

	Strongly Disagree (1)	(2)	(3)	(4)	Strongly Agree (5)
1. I think that I would like to use this program frequently.					
2. I found the program unnecessarily complex.					
3. I thought the program was easy to use.					
4. I think that I would need the support of a technical person to be able to use this program.					
5. I found the various functions in this program were well integrated.					
6. I thought there was too much inconsistency in this program.					
7. I would imagine that most people would learn to use this program very quickly.					
8. I found the program very cumbersome to use.					
9. I felt very confident using the program.					
10. I needed to learn a lot of things before I could get going with this program.					

Scoring:

For odd numbered items (i.e., 1, 3, 5, 7, 9), the score contribution= scale rating – 1
For even numbered items (i.e., 2, 4, 6, 8, 10), the score contribution= 5 –scale rating

The scores are summed and the sum of scores is multiplied by 2.5 to obtain the SUS score. Scores can range from 0-100. A score >68 is considered above average.

References:

Brooke J. SUS: a quick and dirty usability scale. In: Jordan PW, Thomas B, Weerdmeester BA, McClelland AL, eds. Usability Evaluation in Industry. Taylor and Francis; 1996: 189-194.

https://www.usability.gov/how-to-and-tools/methods/system-usability-scale.html

Supplementary Table 3. CONSORT 2010 checklist for reporting a randomized trial

Section/Topic	Item No	Checklist item	Reported on page No
**Title and abstract**			
	1a	Identification as a randomized trial in the title	1
	1b	Structured summary of trial design, methods, results, and conclusions (for specific guidance see CONSORT for abstracts)	1
**Introduction**			
Background and objectives	2a	Scientific background and explanation of rationale	2
	2b	Specific objectives or hypotheses	2
**Methods**			
Trial design	3a	Description of trial design (such as parallel, factorial) including allocation ratio	3
	3b	Important changes to methods after trial commencement (such as eligibility criteria), with reasons	N/A
Participants	4a	Eligibility criteria for participants	3
	4b	Settings and locations where the data were collected	3
Interventions	5	The interventions for each group with sufficient details to allow replication, including how and when they were actually administered	3–4
Outcomes	6a	Completely defined pre-specified primary and secondary outcome measures, including how and when they were assessed	4–5
	6b	Any changes to trial outcomes after the trial commenced, with reasons	N/A
Sample size	7a	How sample size was determined	N/A
	7b	When applicable, explanation of any interim analyses and stopping guidelines	N/A
Randomisation:			
Sequence generation	8a	Method used to generate the random allocation sequence	3
	8b	Type of randomization; details of any restriction (such as blocking and block size)	3
Allocation concealment mechanism	9	Mechanism used to implement the random allocation sequence (such as sequentially numbered containers), describing any steps taken to conceal the sequence until interventions were assigned	3
Implementation	10	Who generated the random allocation sequence, who enrolled participants, and who assigned participants to interventions	3

Blinding	11a	If done, who was blinded after assignment to interventions (for example, participants, care providers, those assessing outcomes) and how	N/A
	11b	If relevant, description of the similarity of interventions	N/A
Statistical methods	12a	Statistical methods used to compare groups for primary and secondary outcomes	5
	12b	Methods for additional analyses, such as subgroup analyses and adjusted analyses	N/A
**Results**			
Participant flow (a diagram is strongly recommended)	13a	For each group, the numbers of participants who were randomly assigned, received intended treatment, and were analyzed for the primary outcome	Figure 1
	13b	For each group, losses and exclusions after randomization, together with reasons	Figure 1
Recruitment	14a	Dates defining the periods of recruitment and follow-up	2
	14b	Why the trial ended or was stopped	N/A
Baseline data	15	A table showing baseline demographic and clinical characteristics for each group	Table 1
Numbers analysed	16	For each group, number of participants (denominator) included in each analysis and whether the analysis was by original assigned groups	Tables 2-3
Outcomes and estimation	17a	For each primary and secondary outcome, results for each group, and the estimated effect size and its precision (such as 95% confidence interval)	Tables 2-3
	17b	For binary outcomes, presentation of both absolute and relative effect sizes is recommended	
Ancillary analyses	18	Results of any other analyses performed, including subgroup analyses and adjusted analyses, distinguishing pre-specified from exploratory	Table 3
Harms	19	All important harms or unintended effects in each group (for specific guidance see CONSORT for harms)	Table 3: smoking outcomes
**Discussion**			
Limitations	20	Trial limitations, addressing sources of potential bias, imprecision, and, if relevant, multiplicity of analyses	7
Generalizability	21	Generalizability (external validity, applicability) of the trial findings	7
Interpretation	22	Interpretation consistent with results, balancing benefits and harms, and considering other relevant evidence	7
**Other information**			
Registration	23	Registration number and name of trial registry	N/A
Protocol	24	Where the full trial protocol can be accessed, if available	N/A

Funding	25	Sources of funding and other support (such as supply of drugs), role of funders	7

© 2023 Krishnan N. et al.
